# Identifying fundamental criteria for eating disorder recovery: a systematic review and qualitative meta-analysis

**DOI:** 10.1186/s40337-017-0164-0

**Published:** 2017-11-01

**Authors:** Jan Alexander de Vos, Andrea LaMarre, Mirjam Radstaak, Charlotte Ariane Bijkerk, Ernst T. Bohlmeijer, Gerben J. Westerhof

**Affiliations:** 1 Centre for eHealth and Well-being Research, University of Twente, Psychology, Health, & Technology, Enschede, The Netherlands; 2 Human Concern Foundation, center for Eating Disorders, Amsterdam, The Netherlands; 3 University of Guelph, Department of Family Relations and Applied Nutrition, Ontario, Canada; 4 Optentia Research Focus Area, North-West University (VTC), Vanderbijlpark, South Africa

**Keywords:** Eating disorders, Recovery, Psychopathology, Psychological well-being, Positive mental health, Meta-analysis, Qualitative research, Systematic review, Positive psychology

## Abstract

**Background:**

Outcome studies for eating disorders regularly measure pathology change or remission as the only outcome. Researchers, patients and recovered individuals highlight the importance of using additional criteria for measuring eating disorder recovery. There is no clear consensus on which additional criteria are most fundamental. Studies focusing on the perspectives of recovered patients show criteria which are closely related to dimensions of positive functioning as conceptualized in the complete mental health model. The aim of this study was to identify fundamental criteria for eating disorder recovery according to recovered individuals.

**Methods:**

A systematic review and a qualitative meta-analytic approach were used. Eighteen studies with recovered individuals and meeting various quality criteria were included. The result sections of the included papers were searched for themes that were stated as criteria for recovery or ‘being recovered’. All themes were analyzed using a meta-summary technique. Themes were labeled into criteria for recovery and the frequency of the found criteria was examined.

**Results:**

In addition to the remission of eating disorder pathology, dimensions of psychological well-being and self-adaptability/resilience were found to be fundamental criteria for eating disorder recovery. The most frequently mentioned criteria were: self-acceptance, positive relationships, personal growth, decrease in eating disorder behavior/cognitions, self-adaptability/resilience and autonomy.

**Conclusions:**

People who have recovered rate psychological well-being as a central criterion for ED recovery in addition to the remission of eating disorder symptoms. Supplementary criteria, besides symptom remission, are needed to measure recovery. We recommend including measurements of psychological well-being and self-adaptability/resilience in future research, such as outcome studies and in routine outcome measurement.

## Plain English summary

In this study, we examined the perspective on criteria for eating disorder recovery among recovered patients. We searched in scientific databases for all published qualitative studies on eating disorder recovery. Eighteen studies were included after meeting rigorous inclusion criteria. The results sections of these studies were analyzed by extracting relevant themes for eating disorder recovery. After calculating effect sizes for the criteria, we found high effect sizes for: self-acceptance, positive relations with others, personal growth, eating disorder remission, self-adaptability, and autonomy, indicating that these are important criteria according to recovered individuals. In addition to the remission of the eating disorder symptoms, dimensions of psychological well-being and self-adaptability/resilience are found as important criteria for eating disorder recovery. This study, among others, shows relevant criteria for eating disorder recovery in addition to the remission of eating disorder symptoms.

## Background

Eating disorders (EDs) are serious mental disorders that impact all facets of people’s lives, including quality of life at home and work, personal functioning, and social life [1–3]. Anorexia Nervosa (AN) has the highest mortality rate of all mental illnesses [4, 5]. Eating disorders are often chronic and refractory [6].

In the last decade, clinical guidelines have been established with treatment options based on evidence (evidence-based care) [7–9]. These treatment options, however, work only for a percentage of patients; for AN in particular, there is no single superior treatment option [10–12]. Effectiveness and efficacy studies, herein called outcome studies, are critical for establishing guidelines for evidence-based care. Outcome studies use measures to examine which treatments are effective, based on the degree of recovery from an ED on certain criteria. There is significant disagreement in the field around the definition of ED recovery, and the relevant criteria that must be present in order to claim “recovery”; see for instance McGilley & Szablewski for an overview [13–18]. As a result, rates of recovery within outcome studies vary widely, ranging from 3% to 96% depending on the criteria used [19]. Recovery is usually measured as the remission of ED symptoms [20]. In a systematic review of 119 patient outcome studies on AN, Steinhausen [20] concluded that remission from all essential clinical symptoms could be considered as recovery; however, he also noted substantial variation in outcome criteria between studies. In a systematic review of predictors of ED outcomes by Vall and Wade [21], over 80% of the 126 included studies reported outcomes based solely on symptom remission [21]. Commonly-used measures were: frequency or absence of binging/purging, change or reaching cut off scores on a questionnaire/interview for measuring ED symptoms (Fairburn & Beglin [22]), changes or remission from overall ED symptoms, or change in BMI or reaching a specific cut off point [21]. In sum, outcome studies generally frame recovery around clinically relevant changes in ED symptoms, or remission.

Simultaneously, a growing body of literature in the ED field highlights that ED symptom change (remission) is not sufficient for understanding, capturing and measuring ED recovery and emphasizes the importance of additional criteria, related to (mental) health, such as quality of life, well-being, psychological, social and emotional functioning [16, 23–26]. This study aims to identify fundamental criteria for recovery from eating disorders focusing on criteria related to clinical symptoms and additional criteria, related to mental health and well-being.

### Mental health: the important role of well-being

Psychologists have lobbied for decades to convey that health is not merely the absence of disease (i.e. symptoms), but also the presence of something positive [27–34]. The emergence of positive psychology, for example, is based on re-focusing the exclusive attention on absence of pathology as a marker for health only, to positive aspects of mental and social functioning as markers for well-being as well [30]. This is in line with the declaration of the World Health Organization (WHO) on mental health: ‘a state of well-being in which the individual realizes his or her own abilities, can cope with the normal stresses of life, can work productively and fruitfully, and is able to make a contribution to his or her community’ (p. 12 [35]). Keyes [36] proposed the ‘complete mental health model’, based on this definition, taking both the absence of psychopathology and the presence of well-being as two related but different aspects of health into account. He did not define health and well-being as a fixed state, but operationalized it as a syndrome consisting of several criteria, where upon people can develop, meeting certain thresholds for optimal well-being [37].

Well-being is theoretically divided into psychological, emotional and social well-being [31, 37, 38]. Psychological well-being (PWB) was conceptualized by Ryff [38] and consists of six key dimensions: self-acceptance, autonomy, environmental mastery, purpose and meaning in life, personal growth and positive relationships with others [34, 38]. This is the model used when we refer to PWB throughout this article. Emotional well-being includes happiness, positive affect and avowed life satisfaction. Social well-being encompasses social contribution, integration, actualization, acceptance and coherence [36]; see for instance [38–40] for an overview of well-being and its theoretical and philosophical background. Recent studies show that psychopathology and well-being are separate but complementary aspects of mental health and reflect two related continua, instead of being opposites on one continuum [29, 37, 40]. In addition, a bi-directional relationship between psychopathology and positive mental health over time is found [41].

The complete mental health model emphasizes the importance of positive functioning for mental health, however, this is widely neglected in research on eating disorders [42]. While several studies have focused on positive mental health in terms of quality of life or subjective well-being, only one study examined all PWB dimensions among eating disorder patients [1, 3, 23, 42–44]. In the study that examined PWB, the authors found that ED patients had impaired PWB compared to a control group [42]. Also, these studies examined the presence of PWB among eating disorder patients, it has not been examined as a criterion for recovery.

In qualitative research examining recovery criteria from eating disorders, there are many recovery themes that are related to the dimensions for well-being. Bowlby and Anderson [45], for instance, found several themes for recovery in a sample of therapists who were recovered from an eating disorder. Most of these themes matched the descriptions of the well-being dimensions. For example, the themes ‘learning to understand and value the self’ matches with the well-being dimensions “personal growth” and “self-acceptance”, the theme “finding purpose and meaning in life” matches with the dimension “purpose” and the theme “developing healthy and meaningful relationships” matches with “positive relationships with others”. In a survey examining criteria for recovery from eating disorders, Noordenbos and Seubring [25] found high consensus between ex-patients and clinicians on all of the proposed 52 statements, divided into five themes: eating behavior, physical, psychological, emotional, and social functioning. However, patients labeled self-esteem, a positive body attitude and expressing emotions as more important, while therapists accentuated eating behavior and physical recovery [25]. Emanuelli and colleagues [26] replicated this study in a sample of patients and clinicians, and concluded that recovery included general criteria (e.g. social, psychological, and emotional) and specific eating disorder criteria (e.g. weight controlling behaviors and evaluation of one’s appearance) [26]. Patients and clinicians agreed on the ranking of importance of most criteria, but patients considered “psychological, emotional, social” and “evaluation of one’s own appearance” criteria as more important for recovery than did clinicians. The researchers did not find weight and weight gain as central criteria for defining recovery [26]. Dawson, Rhodes and Touyz [24] used a different approach, and conducted an extensive Delphi study with ED professionals to determine criteria for recovery from Anorexia Nervosa (AN). They also concluded that, in addition to the minimal criteria (i.e., weight restoration and symptom reduction), psychological and quality of life measures should be part of the definition for AN recovery.

While these studies show the importance of additional criteria, they have several limitations, making it difficult to understand which criteria are most fundamental besides the ED pathology based criteria (remission). Noordenbos and Seubring [25] and Emmanuelli and colleagues [26] used a pre-fixed set of statements, making their study susceptible to missing criteria which might have been endorsed by ex-patients or clinicians had they been articulated in the design of the study. Several qualitative studies show criteria for recovery that were not present in the consensus studies with the pre-fixed statements [25, 26], such as improved self-acceptance, identity development, feelings of purpose and meaning in life, self-management and empowerment [45–47]. Other studies only focused on AN or did not take the perspective of recovered individuals into account. The importance of exploring the perspectives of those with lived experience on their recovery cannot be understated in this regard. Studies have shown that the orientation of patients towards recovery can change over time and during treatment [48, 49].

In conclusion, outcome studies tend to follow criteria for recovery that are based on changes in ED symptoms (remission), rather than aiming to ascertain health and well-being. It remains inconclusive which recovery criteria should be considered as fundamental. We argue that knowledge from individuals who have recovered from an ED should be leading and incorporated into the establishment of fundamental criteria for recovery. Qualitative studies examining the personal experience from recovered individuals highlight the importance of taking additional recovery criteria into account, which are closely related to the dimensions of well-being. However, the results of these qualitative studies have never been systematically reviewed. Responding to this knowledge gap, we carried out a systematic review and meta-analysis of existing qualitative studies of ED recovery.

The aim of this study is to identify fundamental criteria for recovery according to recovered individuals by performing a qualitative meta-analysis. Qualitative meta-analysis can be explained as the aggregation of studies to discover the essential elements of a phenomenon, and translating these results into a more comprehensive description or clear end-product [50, 51]. An integrative interpretation of findings from multiple qualitative studies is therefore more substantive than those resulting from individual investigations [50, 52]. To our knowledge, this is the first study to use a qualitative meta-analysis to further identify fundamental criteria for ED recovery over all ED types, among people who were considered recovered.

## Method

### Search strategy and selection of studies

Guidelines from the PRISMA statement for reporting systematic reviews were used for the search strategy [53]. The first step was to perform a systematic search in two electronic databases, Medline and PsycInfo (final search date 04–02-2016). Terms were searched within all fields. There was no limitation for the year in which the study was published. The main search terms were (Recovery OR Recovered) AND (Eating Disorders OR Anorexia Nervosa OR Bulimia Nervosa OR Binge Eating Disorder) AND (Qualitative), resulting in 238 hits from PubMed and 403 hits from PsycInfo (with a subselection “qualitative studies”).The second step was an additional search in which the reference list of two comprehensive qualitative studies of eating disorder recovery [45, 46] were screened. The third step was to screen all articles in the Google Scholar search engine that had cited [45, 46] (search date: 06–02-2016). Duplicates were removed as follows: 103 duplicates between PsycInfo and PubMed, 5 duplicates between study [45] and [46] and 49 duplicates between the first (PsycInfo and PubMed) and the additional search. In total 630 unique studies remained for screening.

The inclusion criteria were studies that 1) reported on the processes or criteria for eating disorder recovery, 2) included recovered individuals, either because they considered themselves to be recovered, and/or the study used a rigorous system to assess recovery, 3) used a qualitative study design, 4) were published in a peer-reviewed journal or edited academic book, and 5) had a rigorous system for ensuring the credibility of data-analysis (i.e. meeting the CASP protocol, see Procedure and analysis). All ED types as defined in the DSM5 [54] were included, since we were interested in overall criteria for recovery for ED patients. Studies which only or primarily included patients who were not considered recovered were excluded, as we were interested in understanding the markers or criteria for recovery, as opposed to future perspectives on recovery from those actively experiencing eating disorders. Unpublished reports and dissertations were not included to avoid studies that have not been peer-reviewed for quality and also to ensure that studies were not duplicating results [55].

**Fig. 1:**
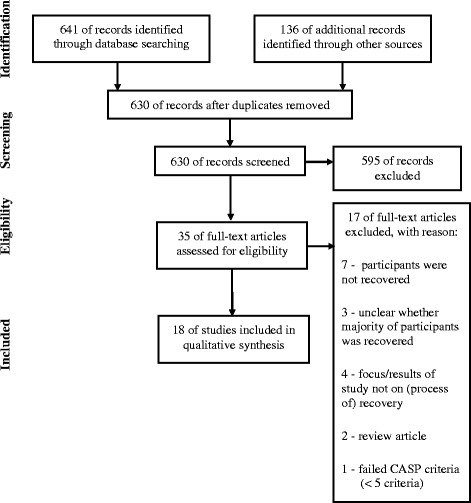
Prisma diagram of study selection

The first and second author screened all eligible studies separately in two phases. In the first phase, selection was based on title and abstract. In the second phase, all selected articles were independently screened by the first two authors based on full text. Inter-rater agreement (kappa coefficient) between authors in the second round of screening was 0.81 (95% CI .68–.91). When there was no agreement, the first two authors discussed decisions to include or exclude studies until agreement was reached. Finally, the reference lists of the included studies were cross-checked on eligible studies. This did not result in extra studies. In total 18 studies were included in the meta-analysis (see Fig. 1).

### Procedure and analysis

A qualitative meta-analysis requires both 1) an assessment of the quality of the studies (i.e. the influence of the method of investigation on the findings) and, 2) results of a more comprehensive explanation of a phenomenon, including its ambiguities and differences found in the primary studies [50].

#### Assessment of the quality of the studies

For the first requirement the Critical Appraisal Skills Programme (CASP) [56] in addition to a complementary rating, was used. The CASP method is a standardized tool to help researchers to systematically examine qualitative studies. CASP is a commonly used method within qualitative meta-analysis, or -synthesis studies to assess credibility, value and relevance of the selected studies [47, 57–59]. In accordance with the CASP method and study [47], the quality of the studies was assessed on 10 themes, and classified as “A”, low risk of bias (studies meeting 9 or 10 of the questions) or “B” moderate risk of bias (studies meeting at least 5 of the questions, but not more then 8). CASP method applies the following 10 criteria: 1) a clear statement of the aims, 2) methodological design is adequate to aims, 3) research design is appropriate to address aims, 4) recruitment strategy is appropriate to aims, 5) data collection in a way that addresses research issue, 6) relationship between researcher and participant is considered, 7) ethical issues are considered, 8) sufficiently rigorous analysis, 9) clear statement of findings, 10) importance of research. Besides the CASP method, a complementary rating for checking credibility was used by dividing studies in “A”, low risk of bias (participants were recovered/in recovery for at least 2 years and recovery was at least self-reported), and “B” moderate risk of bias (participants were recovered/in recovery for less than 2 years, or it was unclear how long participants were recovered and/or it was unclear whether recovery was self-reported). Combining both ratings resulted in 4 possible categories: 1) “A/A”, low risk of bias, 2) “A/B” and, 3) “B/A”, moderate risk of bias, and 4) “B/B” substantial risk of bias.

#### Analysis of criteria for recovery

For the second requirement, a meta summary technique described by Sandelowski & Barroso [60] was used. In contrast to meta-synthesis analysis, this method allows for extracting themes and an evaluation of their frequencies [50, 60]. The following strategy was used: 1) extract relevant themes from each study, 2) reduce these themes into abstract findings and 3) calculate effect sizes. First, the result sections of the included papers were searched for themes that were stated as criteria for recovery or “being recovered”. Themes that were included were: themes that were stated by all participants and themes endorsed by an unknown number of participants, but wherein the theme was part of a main category. For instance, in one study [46], it was unclear how many respondents endorsed on the theme “sense of self-worth”, this theme was, however, part of a main category in the results, “discovering and reclaiming self as good enough” and therefore included. Themes that specifically addressed aspects of the process of recovery (i.e. how long it took, development, stages) were excluded, as were themes that were part of the first or initial phases of a recovery process, since we were interested in criteria which are present when people are fully recovered. The themes were identified independently by the first and second author and stored in their original content. To obtain one dataset for the second step (abstract findings), results were first discussed per study for half of the included articles. For the other half of the studies, the data set of the first author was used by the second author to look for further differences in themes. Differences in found statements were discussed until agreement was reached. This resulted in a dataset with 346 statements which was audited by the third and fourth author.In the second step, the reduction into abstract findings, the labels were established. Eating disorder pathology was divided in three sub labels (behavior/cognitions, body evaluation and physical functions). For the additional themes, the well-being dimensions were used since they seem to relate closely to the themes that are described in qualitative research on eating disorder recovery. The following additional labels were used; emotional, psychological and social well-being with their underlying dimensions as stated in earlier work [31, 36, 38]. Also, a “miscellaneous” label was used for criteria that did not fit into one of the other labels. All themes were read carefully by the first two authors to examine whether they could be labeled corresponding the concept labels. Some of the well-being dimensions were very strictly or narrowly described in the literature [31, 37, 38], A minor adjustment in the description of three labels was necessary for the purpose of labeling the themes (see Table 1 for the adjustments). Then, all 346 original themes were labeled separately by the first and fourth author. Inter-rater agreement (kappa coefficient) for the labeling process between the authors was .81 (95% CI .77–.86) before discussion.

**Table 1:** Labels

Health criteria	Description
1. Eating disorder pathology	
ED behavior/cognitions	Improvement/absence of ED related behavior (bingeing/purging, slimming,) and cognitions (more relaxed/normal thoughts/affect regarding food/weight/exercising).
ED body evaluation	More relaxed regarding body/weight (satisfaction/evaluation).
ED physical functions	Improvement in BMI and/or other physical functions.
2. Emotional well-being	
Avowed happiness	Feeling happy, feeling joy, enjoyment.
Positive affect	Feeling cheerful, in good spirits, calm, and peaceful, satisfied, and full of life.
Avowed life satisfaction	Feeling satisfied with life in general or specific areas of one’s life.
3. Psychological well-being	
Self-acceptance	Holding positive attitudes towards oneself and past life and conceding and accepting varied aspects of self, holding a compassionate attitude towards self. ^*a*^ *Having self-respect. Having feelings of self-worth or self-esteem/confidence. Taking self-care*.
Environmental mastery	Exhibiting the capability to manage a complex environment, and the ability to choose or manage and mould environments to one’s needs.
Positive relationships with others	Having warm, satisfying, trusting personal relationships and being capable of empathy and intimacy and being open and personal to others.
Personal growth	Showing insight into one’s own self and potential, having a sense of development, and being open to new and challenging experiences. ^*a*^ * Identity formation/integration: Having a sense of integration of several/all aspects of self and or formation of (healthy/autonomous) aspects of self*.
Autonomy	Exhibiting a self-direction that is often guided by one’s own socially accepted and conventional internal standards and resisting unsavory social pressures. ^*a*^ * Self-determination, independence, and the regulation of behavior from within* [81]*. Autonomy as used in self-determination theory means acting with the experience of choice* [39].
Purpose in life	Holding goals and beliefs that affirm one’s sense of direction in life and feeling that life had a purpose and meaning.
4. Social well-being	
Social contribution	Feeling that one’s own life is useful to society and that the output of one’s activities is valued by or valuable to others.
Social integration	Having a sense of belonging to a community and deriving comfort and support from that community.
Social actualization	Believing that people, social groups, and society have potential and can evolve or grow positively.
Social acceptance	Having a positive attitude towards others while acknowledging and accepting people’s differences and their complexity.
Social coherence	Being interested in society or social life, and feeling that society and culture are intelligible, somewhat logical, predictable, and meaningful.
5. Miscellaneous labels	
Self-adaptability/resilience	Copingstrategies/resilience/empowerment/willpower/persistance/emotion-regulation, (Healthy) strategies to cope with emotions and difficult life situations.
Spiritual integration	Having a sense of being part of, or in contact with a higher power (Universe, God, Jesus, other) and deriving comfort and support from that. Exercises/activities that promote this: meditation, going to Church, praying etc.

Note: well-being descriptions are published earlier in [29, 31], ^*a*^ = added descriptions to the original labels

#### Interpretation of results

During the discussion, the miscellaneous label could be split into two sub labels (self-management/resilience and spiritual integration). Themes that were part of the discussion were “social contribution” versus “purpose and meaning in life”. The theme “Helping others”, for instance was sometimes explained as a new purpose for participants, but it is also a form of social contribution. Other things that were discussed were; “Identity integration” as part of “personal growth” or as a separate label, and “self-adaptability/resilience” as a part of “autonomy” or as a separate label. See Table 1 for an overview of the final list regarding the labels and descriptives.In the third step, frequency and intensity effect sizes were calculated for all labels. The frequency effect size shows how frequent labels are mentioned across studies and is calculated by dividing the number of studies containing the same finding by the total number of studies [60]. Labels were indicated as strong evidence for ED recovery criteria, when they were reported by at least 75% of the primary studies, as substantial evidence when they were reported by 50% to 75% of the primary studies, as moderate evidence when they were reported by 25% to 50% of the primary studies, and as insufficient evidence if less than 25% of the studies reported on a dimension. Although these cut-off points are rather arbitrary, we decided to use quartiles as cut off for ease of interpretation and pragmatic value for those seeking evidence on recovery criteria.

The intensity effect size gives a clear measure for how fundamental recovery criteria are compared to each other. The intensity effect size is calculated as the number of findings for a criteria produced in all studies, divided by all findings [60]. To examine possible effects of the methodological quality of the studies on the results, differences in outcomes on the intensity effect size between low risk of bias studies (A/A) and substantial risk for bias studies (B/B) were tested using a proportion significance test (χ^2^ test for homogeneity).

## Results

### Descriptives

See Table 2 for an overview of the included studies and quality rating. The 18 included studies covered 286 participants (269 women and 17 men), with an average age of 30.2 years (SD = 7.3 years). One hundred-sixty-three participants had been diagnosed with AN, 25 participants had been diagnosed with Bulimia Nervosa (BN), 18 participants had a history of both AN and BN diagnosis over their life course, 8 participants had been diagnosed with Binge Eating Disorders (BED) and 13 participants had been diagnosed with an Eating Disorder Not Otherwise Specified (EDNOS). The average duration of the eating disorder was 8.2 years (*SD* = 5.1, study number: 1,3,6,11,16) with a minimum length of 1.5 years and a maximum length of 44 years (study number: 1,6,11,16,17). The average length of recovery was 9.1 years (SD = 6.1 years, study number: 1,3,6,11), with a range of 1 year to 35 years (study number: 1,2,6,7,8,11,13,17). However, for many studies this was unknown.

**Table 2:** Summary of included studies

Nr	Study	Country	Diagnosis	N of Participants	Study focus	Ethics approval	Data collection	Data analysis	Credibility	Recovery criteria	Minimalrecovery length	Quality rating
1	[74]	Australia	AN	8	Process of recovery chronic AN	Yes	Open ended interviews (face to face)	Narrative inquiry	Member-check with each participant, cross-check by two authors	Self-reported, Bardone-Cone criteria (2010)	5 years	A/A
2	[46]	Canada	AN	9	Recovery from AN	Unknown	In depth open ended interviews	Grounded theory approach	Review process by 3 individuals	Self-report	3 years	A/A
3	[85]	U.S.	AN, BNEDNOS	17	Athletes’ ED recovery experiences	Yes	Semi structured interviews	Content analysis procedure	Reviewing of coding structure by two colleagues, discussion until agreement	DSM-IV-TR criteria	3 months	B/B
4	[75]	Several	AN	3	Influences on the process of recovery from AN	No	Purposive/extreme sampling to identify published narratives	Framework approach to qualitative analysis	Coding by two authors, discussion until agreement	As described by the personal account	Unknown	B/B
5	[86]	Sweden	BN	5	Experiences of recovery from AN	Yes	Open ended interviews (semi-structured)	Narrative interviews with qualitative content analysis	Added quotations from participants to results	Self-reported (being healthy)	2 years	A/A
6	[87]	U.K.	AN	15	Views of recovering from AN	Yes	Semi structured interviews (in-depth, by phone)	Interpretative phenomenological analysis	Discussion between authors	Self defined recovery/recovered	No requirement	B/B
7	[88]	U.S.	AN	22	Perspectives of recovered individuals on ED, recovery and social support	Yes	Interviews	Generic qualitative description analysis	Separately coded by two authors, comparing initial codes and consensus seeking. Member checking with participants	Measured: eating screen	1 year	A/B
8	[89]	Brazil	AN	15	Factors involved in the outcome of AN	Yes	Ethnographic Face to face interviews (semi-structured)	Grounded theory approach	Analyzing separately, calculating inter-rater agreement	Reported by self, family member and assistant MD	5 years	A/A
9	[90]	Norway Sweden	AN, BNEDNOS	15	Experiences of life after recovery	Yes	Open ended interviews (semi-structured)	Phenomenological approach	Analyzed by three different teams and discussion until consensus	Self-report (experienced recovery or marked improvement)	Unknown	A/B
10	[91]	Sweden	AN	58	Patient perspectives of AN recovery	Yes	Interview	Content analysis procedure	Separate analysis by three researchers, calculating inter-rater agreement	Clinical assessment (DSM-III-R)	Unknown	B/B
11	[45]	U.S.	AN, BN	13	Exploring ED recovery	Yes	Semi structured interviews	Phenomenological approach	Addressing researcher bias, authors independently reading transcripts, and discussing emergent themes, included extended quotations	Self-report	6 years	A/A
12	[79]	Israel	AN	18	Patients perspective of recovery from AN	Yes	In depth semi-structured interviews	Phenomenological approach	Adding quotes to results	DSM-IV	5 years	A/A
13	[92]	Canada	BED	6	Recovery from binge eating disorder	Unknown	Two interviews (unstructured and structured)	Grounded theory approach	Audit by second author	Self-reported, DSM-IV (objective measure)	6 months	A/B
14	[93]	U.K.	AN	6	The patients perspective of recovery from AN	Unknown	Interview	Case descriptions	Unknown	Assessment	Unknown	B/B
15	[94]	U.S.	AN, BN	3	Rethinking recovery	Yes	Semi structured interview	Interpretative biographical method	Working with performance texts	Self-identified as recovered	Unclear (well into process of recovery)	B/B
16	[95]	Sweden	AN, BNEDNOS	14	Patients perception having recovered from an ED	Yes	Semi structured interviews	Phenomenological approach	second author scrutinized statements in relation to conceptions and categories	Self-identified as recovered at 1 year follow-up	Unclear	A/B
17	[96]	Australia	AN, BN	20	Experiences of developing and recovering from ED	Yes	Open ended interviews (semi-structured)	Live history interviews	two researchers reading and making margin notes	Self-reported	3 years	A/A
18	[80]	Canada	AN	12	Understanding journey of recovery from AN	Yes	Interviews	Feminist grounded theory	confirm/refine explication of emerging theory by participants	Self-identified as recovered	Unknown	B/B

### Criteria for eating disorder recovery

See Table 3 for the intensity and frequency effect sizes of the criteria for recovery. The frequency effect sizes show strong evidence for positive relationships with others (100%), self-acceptance (88.9%), autonomy (83.3%), personal growth (77.8%), improved ED behavior/cognitions (77.8%), self-adaptability/resilience (77.8%). Substantial to moderate evidence was found for improved body evaluation (55.6%), social contribution (50%), purpose and meaning in life (38.9%), spiritual integration (33.3%), improved (ED) physical functioning (27.8%) and positive affect (27.8%). Insufficient evidence was found for happiness (22.2%), avowed life satisfaction (22.2%), environmental mastery (11.1%), social acceptance (11.1%), social integration (11.1%), social actualization (0%) and social coherence (0%).

**Table 3:** Meta-analysis: Intensity and frequency effect sizes of ED recovery criteria

Recovery Criteria			All (*N* = 18)	A/A (*N* = 8)	B/B (*N* = 8)		
	Evidence for recovery	Frequency effect size	Intensity effect size	Intensity effect size	Intensity effect size	χ^2^	*P* (2-sided)
Self-acceptance	Strong	88.9%	15.3%	17.6%	13.8%	.679	.486
Positive relationsships with others	Strong	100%	12.7%	13.4%	14.6%	.070	.791
Personal growth	Strong	77.8%	12.7%	18.5%	8.5%	**5.432**	**.020**
Decrease in ED behavior/cognitions	Strong	77.8%	12.4%	9.2%	12.3%	.603	.437
Self-adaptability/resilience	Strong	77.8%	9.2%	9.2%	7.7%	.082	.774
Autonomy	Strong	83.3%	7.8%	8.4%	9.2%	-	.791^*^
Social contribution	Substantial	50%	6.9%	6.7%	6.9%	.004	.950
Improved (ED) body evaluation	Substantial	55.6%	5.8%	1.7%	6.2%		
Spiritual integration	Moderate	33.3%	2.9%	.8%	6.2%	**-**	**.037** ^*^
Purpose & meaning	Moderate	38.9%	2.9%	3.4%	3.1%	.016	.899
Improved (ED) physical functioning	Moderate	27.8%	2.6%	4.2%	1.5%	-	.264^*^
Happiness	Insufficient	22.2%	1.7%	.8%	1.5%	-	1.000^*^
Positive affect	Moderate	27.8%	1.7%	2.5%	.8%	-	.351^*^
Other	-	33.3%	1.7%	.8%	2.3%	-	.623^*^
Avowed life satisfaction	Insufficient	22.2%	1.2%	.8%	.8%	-	1.000^*^
Environmental mastery	Insufficient	11.1%	.9%	.8%	1.5%	-	1.000^*^
Social acceptance	Insufficient	11.1%	.9%	-	2.3%		.247^*^
Social integration	Insufficient	11.1%	.9%	.8%	.8%		1.000*
Social actualization	Insufficient	- -	-	-	-	-	-
Social coherence	Insufficient	- -	-	-	-	-	-

*Note:* Frequency effect size: Total *N* of studies divided by *N* of studies containing a criteria * 100, Intensity effect size: *N* of found criteria produced in all studies, divided by all found criteria in all studies * 100, χ^2^ test of homogeneity (differences in two proportions), ^*^ *p* was calculated by Fisher’s Exact test for violation of the minimal sample size of the χ^2^ test

Examining the effect sizes of the overall mental health dimensions; psychological well-being accounted for 52.3% of all recovery criteria, eating disorder pathology for 20.8%, self-adaptability/resilience and spiritual integration for 13.8%, social well-being for 8.6% and emotional well-being for 4.6%. Examining the intensity effect sizes of the underlying eating disorder pathology criteria; improved ED behavior/cognitions accounted for 12.4% of the whole sample, improved body evaluation for 5.8% and physical improvement for 2.6%. Improved behavior/cognitions were described in the original studies in several ways. Recurring themes were; returning to a normal eating pattern, no weight phobia, or ending the obsession with weight/food. Physical improvement was primarily about weight recovery and improvement of physical complications.

### Testing risk of bias

**Fig. 2:**
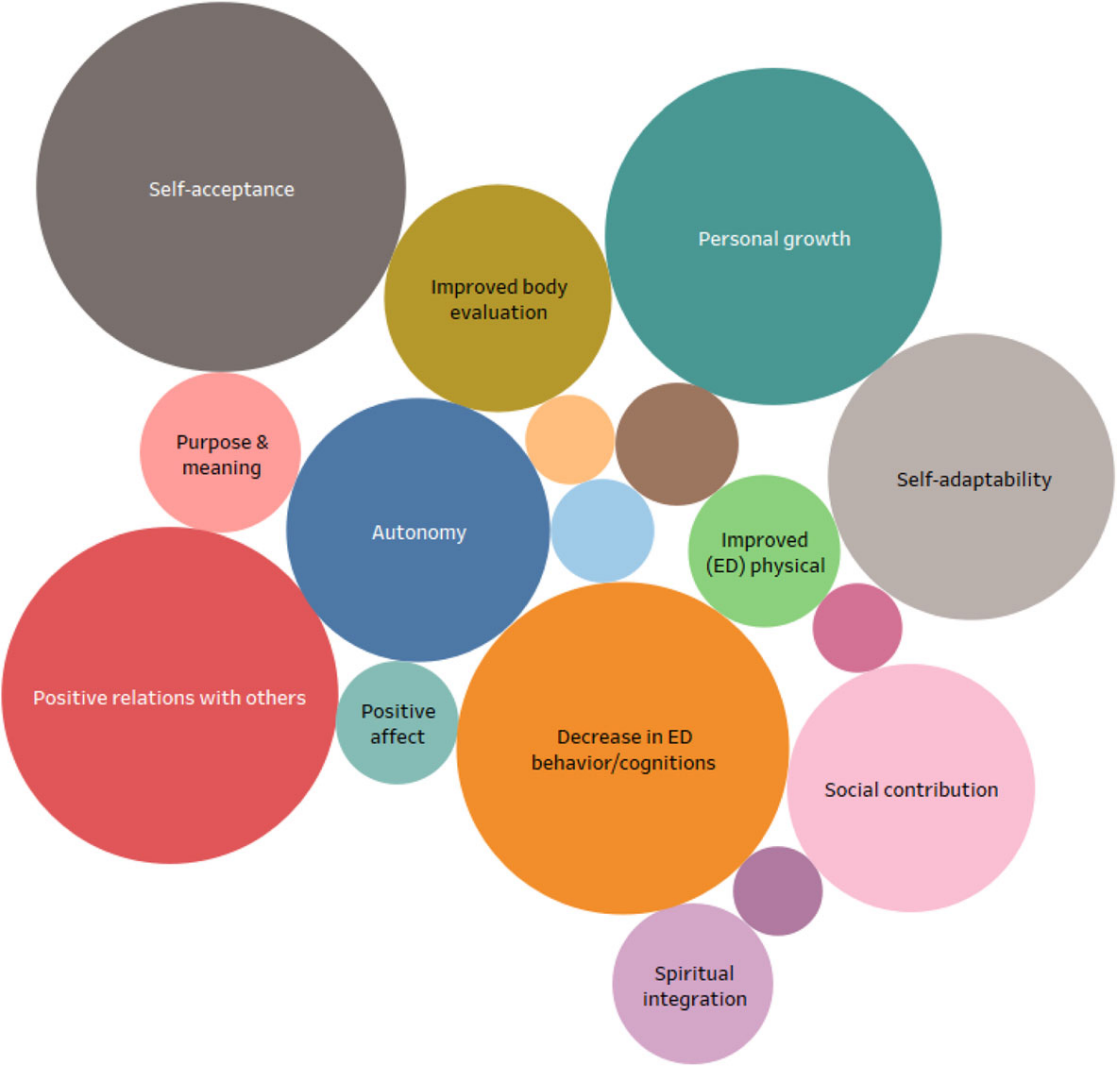
Intensity effect sizes of criteria for recovery. Circles represent criteria for recovery and are based on the intensity effectsizes. The larger the circle, the larger the intensity effectsize. Circles that are labeled with a text have moderate, substantial or strong evidence for a recovery criteria. Circles that are not labeled with a text are the remaining criteria

Eight studies had an “AA” status and 8 studies a “BB” status. Except for the criteria “personal growth” and “spiritual integration”, no differences in proportions of the intensity effect sizes were found between “AA” and “BB” studies (see Table 3). Only two studies had a moderate indication for bias (A/B, or B/A) status and could not be used for testing significance because of the low sample size (Fig. 2).

## Discussion

Criteria for recovery were examined using a qualitative meta-analytic approach. Studies were selected that examined the personal experiences of recovered individuals.

### Fundamental recovery criteria

The aim of this study was to identify fundamental criteria for ED recovery according to recovered individuals. Several health dimensions besides symptom remission were found that should be considered as fundamental criteria of eating disorder (ED) recovery. Large frequency effect sizes, indicating strong evidence, were found for the following six criteria: positive relationships with others, self-acceptance, autonomy, personal growth, improved ED behavior/cognitions and self-adaptability/resilience. Further, substantial to moderate evidence was found for the following six criteria: improved body evaluation, social contribution, purpose and meaning in life, spiritual integration, improved physical functioning and positive affect. At last, insufficient evidence was found for the following seven criteria: happiness, avowed life satisfaction, environmental mastery, social acceptance, social integration, social actualization and social coherence. These results show a clear perspective of the relevant criteria from the perspective of people who have experienced recovery. While remission of ED pathology is considered important, many criteria were about psychological well-being (PWB). Moreover, PWB was mentioned more (52.3% of all criteria) than the remission of ED pathology (20.8% of all criteria) as a marker for recovery.

These results underscore the conclusions of earlier work about the importance of including psychological dimensions in definitions of ED recovery [17, 26, 61]. PWB is not about happiness or positive affect, but explained as living a good life, with purpose and meaning, growing towards optimal functioning and self-realization [39, 62]. The philosophical roots of PWB lead back to Aristotle’s formulation about the virtues life. The essence of this Greek philosophy is to know yourself and to become what you are [62]. Many themes in the studies were about finding a new or ‘healthy’ identity and developing self-insight and self-acceptance. Our results suggest that the underlying dimensions of PWB should be considered as fundamental aspects of ED recovery, perhaps even important to focus on during treatment than the abatement of symptoms. A focus on well-being in treatment has been suggested earlier for other psychiatric disorders by Fava and others [32, 41]. It is noted that Parloff and colleagues already suggested in 1954 that the goals of psychotherapy were not necessarily the reduction of symptoms, but increased personal effectiveness [32]. Several therapies have been developed focusing on PWB [63–68]. PWB is further related to work productivity, physical and overall mental health, and care consumption, even when controlling for symptoms of mental illness [37, 69, 70]. It can also improve the quality of life for psychiatric patients, and the change to recover on symptoms and decrease the risk of relapse [69, 71].

Environmental mastery was the only PWB dimension that showed insufficient evidence. However, environmental mastery could be considered to be an aspect of self-adaptability. Self-adaptability is defined broader, taking social and emotional adaptability into account. If the description of environmental mastery was described more broadly, taking all aspects of self-adaptability into account, this probably would have been found as evidence for a criterion for ED recovery. Limitations in the first WHO definition of health have recently led to a new definition of health, described as the ability to adapt and to self-manage, in the face of social, physical and emotional challenges [72, 73]. The importance of self-adaptability/resilience as a criterion for ED recovery, fits this recently proposed definition of health [73]. In addition, Ryff stated that PWB is fundamentally anchored in how individuals face the challenges of life [62]. It is noted that “being recovered” is certainly not achieving a perfect state on the found criteria. It is explained as a unique and self-determined process by recovered individuals, without a clear endpoint [46, 74–79]. A new definition for ED recovery based on the latest definition of health and the results of this study could be: recovery from an ED is the ability to adapt and to self-manage in the face of social, physical and emotional challenges with an overall tendency towards growth in psychological well-being and adequate symptom remission (for instance as operationalized by Bardone-Cone et al. [18]). ED patients reported an overall impairment in PWB in a controlled study, which was not necessarily dependent on the presence of high levels of symptom severity, suggesting that PWB does not simply correspond to the absence of pathology [42]. Well-being and pathology as two different but related aspects of health has been well validated in several samples of the normal population and in patients [31, 36, 41].

“Recovery” may also indicate both a process and a state [32]. For eating disorder recovery, criteria also occupy a tenuous place between facilitators of recovery and criteria for demonstrating recovery in the literature. It is not always clear whether these themes are offered as requirements for ascertaining the degree to which someone is recovered or as facilitators to achieve recovery. In outcome studies, most themes, from changes in BMI to improvement in self-esteem, are used both as predictor variables and outcome variables; see for instance Vall and Wade [21]. One of the conclusions of a recent meta-synthesis was that the presence of supportive relationships is an important facilitator for recovery [47]. Recovery in the qualitative sense is often described as a process or journey [74, 75, 80]; and yet, what we need in a clinical sense is criteria to gauge and compare outcomes (see also Rosenvinge and Pettersen [17], p. 1). We argue that recovery dimensions that remain important aspects for individuals’ health, such as positive relationships, are operationalized as criteria for recovery, in accordance with health and well-being definitions [37, 81]. It is likely that these criteria, related to well-being, are also important as criteria for recovery for other pychiatric disorders, such as depression. In a sample of patients with depressive symptoms, it was found that not only psychopathology improves, but also that PWB increases during treatment [82]. In another outcome study it was found that many patients with depressive symptoms improved either on psychopathology, or on the well-being dimensions and not on both, suggesting that both are important to measure in outcome studies and should be considered as criteria for recovery for psychiatric disorders [83].

### Limitations

Although a qualitative meta-analytic approach allows for a more comprehensive explanation of a phenomenon than the individual qualitative studies explain, there are several limitations concerning this study. First, the presented methods and results were influenced by the methodology of the primary studies and their findings. Some of the primary studies failed to provide sufficient details about the background of the participants, used methods and/or results. It is also unclear how different systems of data analysis have formed the results in the primary studies. By examining differences in outcomes between low risk of bias studies and risk of bias studies we tried to minimize the risk of bias. Second, that some hypothesized dimensions were or were not supported does not necessarily depend only on the studies and participants, but also may be a flaw in inadequate thematic analysis or misclassification of themes. We tried to address possible classification bias by independent analysis and calculating an interrater agreement. Third, this study shows frequencies, constituting the importance of recovery criteria, but fails to show the contradictions between studies, including, but not least, that to claim that those in the study were recovered, they had to determine provisional criteria for recovery, which differed significantly between studies. The method of this study did not allow to examine differences in criteria between type of eating disorder. Most primary studies focused on either, AN, or all eating disorder types, which makes it difficult to divide results into ED type groups. Further research could focus on differences in well-being criteria between ED types. In a study examining the dimensions of psychological well-being among ED patients, differences in severity were found [42]. Compared with a control group, patients with BN had greater impairment on all psychological well-being scales, whereas patients with BED showed greater impairment on only three scales and patients with AN on only two scales. It is possible that improvement on the several well-being dimensions has a different priority depending on the ED type [42]. Also, the search strategy was quite narrow, with a lack of synonyms for “recovery” or “recovered”, such as “remission”, “rehabilitation”, “restoration”. However, we argue that these synonyms are not used regularly in qualitative ED recovery studies examining the view of patients or recovered individuals. In fact, in the reference-check, no suitable other studies were found using these synonyms. At last, this study examined criteria for being recovered among people who were considered recovered. Further research should examine how these recovery criteria develop and influence each other during the recovery process.

## Conclusions

We conclude that psychological well-being and self-adaptability are core aspects of recovery in addition to remission of ED symptoms. A focus in treatment on these health dimensions seems therefore important to achieve recovery. Whether someone is recovered or not remains a question primarily to be answered by the patient her/himself. However, to find best treatment options, researchers and clinicians need to measure the most fundamental criteria for recovery. This study, among other studies [16, 17, 25, 26, 47, 84], provides a further direction to understand which criteria are most important to measure. Developing and validating instruments that measure recovery on these fundamental criteria is warranted. It is also advised to establish an international standard or guideline on how to measure ED recovery outcomes and which instruments to use, so that we might be able to compare treatment outcomes in the near future.

## Abbreviations

AN: Anorexia Nervosa
BED: Binge Eating Disorder
BN: Bulimia Nervosa
ED: Eating Disorder
EDNOS: Eating Disorder Not Otherwise Specified
PWB: Psychological Well-being
